# Clinical, Radiological and Pathological Characteristics Between Cerebral Small Vessel Disease and Multiple Sclerosis: A Review

**DOI:** 10.3389/fneur.2022.841521

**Published:** 2022-06-24

**Authors:** Bijia Wang, Xuegang Li, Haoyi Li, Li Xiao, Zhenhua Zhou, Kangning Chen, Li Gui, Xianhua Hou, Rong Fan, Kang Chen, Wenjing Wu, Haitao Li, Xiaofei Hu

**Affiliations:** ^1^Department of Neurology, First Affiliated Hospital, Army Medical University (Third Military Medical University), Chongqing, China; ^2^Department of Neurosurgery, First Affiliated Hospital, Army Medical University (Third Military Medical University), Chongqing, China; ^3^Department of Radiology, First Affiliated Hospital, Army Medical University (Third Military Medical University), Chongqing, China

**Keywords:** cerebral small vessel disease, multiple sclerosis, magnetic resonance imaging, pathological features, review

## Abstract

Cerebral small vessel disease (CSVD) and multiple sclerosis (MS) are a group of diseases associated with small vessel lesions, the former often resulting from the vascular lesion itself, while the latter originating from demyelinating which can damage the cerebral small veins. Clinically, CSVD and MS do not have specific signs and symptoms, and it is often difficult to distinguish between the two from the aspects of the pathology and imaging. Therefore, failure to correctly identify and diagnose the two diseases will delay early intervention, which in turn will affect the long-term functional activity for patients and even increase their burden of life. This review has summarized recent studies regarding their similarities and difference of the clinical manifestations, pathological features and imaging changes in CSVD and MS, which could provide a reliable basis for the diagnosis and differentiation of the two diseases in the future.

## Introduction

Cerebral small vessel disease (CSVD) belongs to a group of diseases involving cerebral arterioles, venules and capillaries. CSVD accounts for about 25% of ischemic stroke, and its etiology remains unclear. The pathogenesis may be related to vascular endothelial injury, hypoperfusion and ischemia, and impaired blood-brain barrier function (1, 2). Currently, CSVD can be divided into 6 categories according to different pathological characteristics: atherosclerosis, cerebral amyloid angiopathy, hereditary vascular diseases [such as cerebral autosomal dominant arteriopathy with subcortical infarcts and leukoencephalopathy (CADASIL), Fabry disease, etc.], inflammatory vascular diseases (such as primary angiitis of the central nervous system (PACNS), Susac syndrome, etc.), venous collagen deposition lesion and other types (3, 4). The imaging features included recent small subcortical infarcts, lacune of presumed vascular origin, white matter hyperintensities of presumed vascular origin, perivascular space (PVS), cerebral microbleed (CMB) and brain atrophy. Some patients may obtain CSVD-related symptoms, but many patients lack symptom and the occurrence of such imaging features appears more problematic within individuals above the age of 50. However, after repeated small vessel events, patients often show abnormal gait, numbness of limbs, cognitive decline and etc., which can seriously result in cognitive dysfunction and physical disability. It has been reported that the etiology of 45% patients with dementia is CSVD, which ranks second only to Alzheimer's disease (3, 5). Therefore, a series of complications caused by CSVD bring immeasurable burden and cost to society.

Multiple sclerosis (MS) is an autoimmune disease characterized by inflammatory demyelination of white matter centered on the central nervous system. The etiology and pathogenesis of MS remains to be unknown. In terms of anatomical structure, MRI showed that the white matter lesions of MS were often distributed around the paracele, subcortex, thalamus, brainstem, and others, showing hyperintensity signal lesions on T2-weighted images (6). MS is an important cause of non-traumatic nervous system dysfunction in young people. Epidemiologically, the incidence of MS shows obvious differences in different regions of the world, and the incidence of MS in Europe and America is higher than that in Asia and Africa. The incidence of MS in females is higher than that in males. Usually, the clinical onset age ranges from 20 to 40 years old, and its clinical manifestations are non-specific. The main symptoms include limb numbness, limb weakness, dizziness, ataxia, blurred vision and others, which are often similar to the clinical manifestations of other inflammatory-related diseases and non-inflammatory-related diseases, thus making them difficult to be distinguished. If patients cannot be correctly diagnosed and treated in the early stage of MS progression, more than half of patients will lose their independent exercise ability after 20 years (7, 8). It not only seriously affects the quality of life of patients, but also brings more medical burden to all sectors of society.

In clinical work, CSVD and MS often have similar characteristics in clinical manifestations, imaging features and pathological changes, which brings many difficulties to the identification, early diagnosis and treatment intervention of clinical diseases, and further delays the effective treatment time window of patients. Therefore, it is helpful to improve our diagnosis, comprehensive evaluation and formulate treatment strategies in clinical work through in-depth understanding of the similarities and differences in clinical symptoms, imaging changes and pathological features of these two diseases. This review intends to summarize and discuss the similarities and differences between CSVD and MS from three aspects, such as clinical features, imaging changes, and pathological features, so as to provide a strong basis for the subsequent clinical diagnosis and treatment.

## CSVD

### Clinical, Pathological, and Imaging Features of CSVD

**Figure 1:**
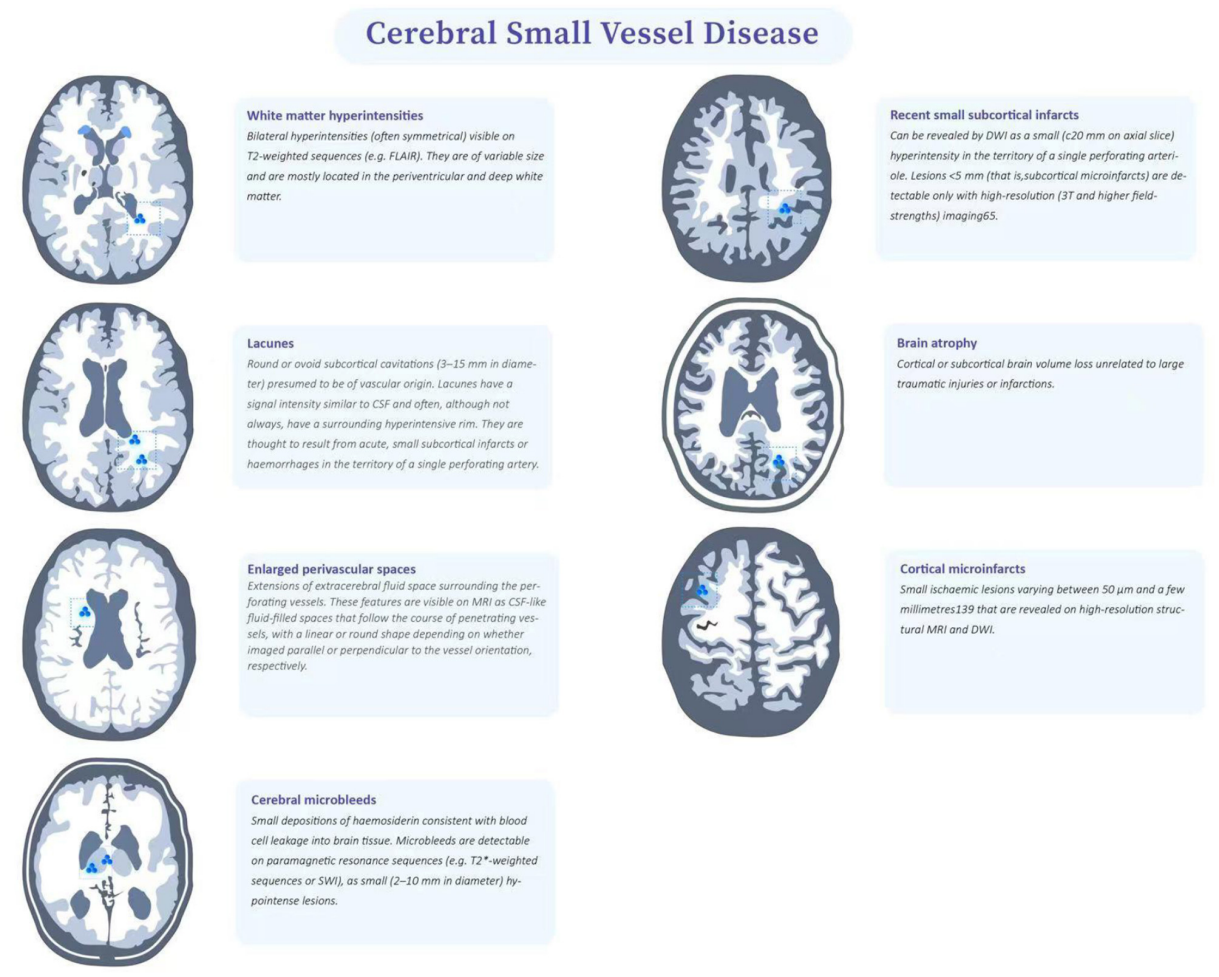
Features of CSVD.

CSVD refers to a series of clinical, imaging, and pathological syndromes resulting from various etiologies affecting small intracerebral arteries and their distal branches, microarteries, capillaries, microvenules, and small veins. The current definition of CSVD is broader and includes not only the small vessels mentioned above, but also the vascular structures within the brain parenchyma and subarachnoid space 2–5mm surrounding these small vessels (9). CSVD is one of the important causes of white matter lesions. Due to the occult onset and mild symptoms, it is difficult to be diagnosed by screening in clinic. However, repeated cerebrovascular events will eventually result in the limb dysfunction and seriously affect the quality of life. Pathologically, cerebrovascular arteriosclerosis often occurs in CSVD, which is highly correlated with age and vascular risk factors (VRFs) (e.g., hypertension) (10). The results of pathological sections indicated that fibrinoid necrosis in the vascular wall, lipohyalinotic degeneration, atherosclerotic plaque, thickening of vessel wall and narrowing of vascular lumen could all promote the formation of cerebral arteriolar sclerosis. The loss of smooth muscle cells in the vessel wall will lead to the dysfunction of vascular autonomic regulation and the formation of microaneurysms. In view of the fact that arteriosclerosis is a systemic pathological process, similar pathological findings exist in target organs with more small blood vessels, such as retina and kidney (5). Although many previous studies on CSVD have made some achievements, the pathogenesis of CSVD remains to be unclear. Imaging can not only assist the diagnosis of CSVD and reflect the degree of involvement, but also help to understand its pathological basis. However, imaging description has individual heterogeneity (3). Therefore, an expert consensus was organized and drafted by the international working group, which classified the imaging features of CSVD as recent small subcortical infarcts, lacune of presumed vascular origin, white matter hyperintensities of presumed vascular origin, perivascular space (PVS), cerebral microbleed (CMB) and brain atrophy (3, 9) (Figure 1).

### Classification of CSVD Based on Imaging

#### Recent Small Subcortical Infarcts

The infarct foci usually smaller than 20mm are considered as the imaging features of recent small subcortical infarcts or acute lacuna infarction. These lesions progress to potential lacunae or white matter hyperintensities without cavities over time, and may also disappear (11). These lacunae are generally round or oval, with a diameter of about 3 to 15mm, and their signal strength is consistent with CSF (3). The blood supply area of perforating arterioles is the predilection site of recent small subcortical infarcts, which can gradually evolve into lacuna and usually appear with white matter hyperintensities in chronic stage, suggesting that lacuna and white matter hyperintensities may have interrelated pathological basis (12). Another distinguishing feature is that in the fluid attenuated inversion recovery (FLAIR), the lesion area can be seen with hyperintensity at the edge, which suggests that the lesion is more likely to be a lacuna than a perivascular space. Multiple perivascular spaces in basal ganglia are called “sieve state,” which is usually related to brain atrophy and brain degenerative disease (3, 5).

#### Lacune of Presumed Vascular Origin

Lacune of presumed vascular origin are generally small cavities that remain in the brain tissue after removal of the necrotic tissue of a subcortical infarction. Most symptoms lack clear corresponding clinical manifestations and may lead to progressive neurological decline such as cognitive decompensation or even vascular dementia (13), balance disorders, gait disturbances, urinary incontinence and affective disorders after several episodes of mild hemiparesis. The cognitive decrements caused by lacunae are dominated by executive function decrements. For motor impairment, an increased number of lacunae is associated with slower gait speed, wider step base and reduced balance; thalamic and frontal lacunae are associated with slower gait speed, reduced stride length and slower gait frequency (14).

#### White Matter Hyperintensities of Presumed Vascular Origin

Frontal lobe and parietal lobe are the predilection sites of WMHs (15), and about 80% of white people over 60 years old suffer from WMHs. According to the scope of WMH, it can be divided into three types: focal, initial fusion and extensive fusion (16, 17). The terminal region of WMHs blood supply is the predilection site of WMHs. In the supratentorial region, it is more common in basal ganglia, corona radiata and centrum semiovale (12). In the brain stem position, it is more found in the center of brainstem (18). Previous studies have shown that the progress of WMHs is related to cognitive impairment, behavioral impairment, gait abnormality and urination disorder, and develops with time (4, 19, 20).

#### PVS

The PVS is an extension of the extracerebral fluid gap surrounding the arteries, small arteries, veins, and small veins. Since the PVS enters from the brain surface or through the brain parenchyma, it can be traced through the lamellar soft meninges (21, 22). As patients age, PVS becomes increasingly evident and especially PVS at the base of the brain (21). Several studies have shown that enlarged PVS is associated with reduced cognitive function (22). The clinical symptoms associated with PVS are still in the exploratory stage. Several cross-sectional studies have found that PVS is associated with reduced information processing speed, reduced executive function, and increased risk of vascular dementia (23). However a meta-analysis that included five large studies found that PVS was not associated with cognitive function in healthy older adults (21). There is limited research on PVS and movement disorders. Previous case reports have suggested that severe striatal area PVS is associated with motor symptoms in Parkinson's disease, possibly because severe PVS affects striatal structure and function, which in turn leads to extrapyramidal symptoms.

#### CMB

SWI (susceptibility weighted imaging) or GRE (gradient recalled echo) is relatively sensitive to the exploration of cerebral microhemorrhage. The lesions are <5mm in diameter on images, but not visible on other MRI sequences or CT (computed tomography, CT) (24). When complicated with chronic hypertension and atherosclerosis, microhemorrhage usually occurs in deep gray matter, and should be distinguished from calcification, normal blood vessels, iron deposition from other causes, hemorrhagic metastasis and brain trauma (3, 25).

#### Brain Atrophy

Brain atrophy is also an important imaging manifestation of CSVD, and ventricular enlargement can be seen on imaging. Usually, the T1 sequence is the most suitable for evaluation, and the corresponding rating scale can also be used (26). However, atrophy caused by large vascular infarction, trauma and other focal lesions is not attributed to brain atrophy caused by CSVD (3).

### Classification of CSVD Based on Pathological Features

#### Cerebral Amyloid Angiopathy

Based on the pathological characteristics of CSVD, cerebral amyloid angiopathy (CAA) is considered to be a vascular disease caused by β-amyloid protein deposition in the middle and adventitia of cortex and pia mater artery. This disease occurs in the white matter of occipital lobe and frontal lobe. Different from systemic amyloidosis, CAA commonly occurs in middle-aged and elderly people, and the proportion of CAA over 55 years old is as high as 10–40% (27). In addition, 80% of AD patients also coexist with CAA lesions. The pathological features of this disease include the occurrence of hemorrhage foci of different sizes in cerebral lobes, subcortex and cortex with time changes, and the signs of subarachnoid hemorrhage in the past (28). Histochemical staining (such as Congo red staining) showed that β-amyloid protein was deposited on the vascular wall, and the thinning and loosening of the vascular wall, vascular dilatation and perivascular inflammation were another important pathological features of CAA (29). Increased vascular fragility is often one of the important reasons for bleeding tendency. Previous imaging studies suggest that chronic hypoperfusion is one of the risk factors of leukoencephalopathy, Therefore, lobar hemorrhage, non-traumatic subarachnoid hemorrhage and WMH are also common imaging manifestations of CAA (30). According to the revised Boston Diagnostic Criteria, the results of imaging examination are suggestive for diagnosis, but autopsy is still needed to make a definite diagnosis (31, 32).

#### CADASIL

CADASIL is an autosomal dominant disease associated with a mutation in the NOTCH3 gene on chromosome 19. The incidence rate reaches 2–4/100000, which has no significant correlation with gender. Clinically, it usually occurs at the age of 30–40 years old, with various clinical manifestations, including migraine, recurrent ischemic stroke, progressive cognitive dysfunction in young people and etc. Pathology suggests that the disease is a non-atherosclerotic CSVD without amyloid deposition (33). CADASIL is the most common genetic factor of subcortical vascular dementia. Under electron microscope, granular osmiophilic material (GOM) was found deposited in the smooth muscle layer of pia mater artery and perforator vessels. This abnormal deposition causes the loss of smooth muscle cells, which leads to the narrowing and even occlusion of the official lumen of small blood vessels (34–36).

The imaging features of the disease show a dynamic evolution process with the age of patients. First of all, WMHs can be found in temporal pole in most CADASIL patients around 30 years old, which is a characteristic different from other microangiopathy. At the age of 40, WMHs gradually progressed to the posterior temporal region, frontal region, parietal region, basal ganglia and thalamus (37). Subcortical U-shaped fibers can be involved, and subcortical lacunar infarction can often be found. The outer capsule and corpus callosum are also involved, which are important landmark lesions of CADASIL. At the age of 50, cerebral microhemorrhage appeared in the white matter area of the patient's brain and gradually developed. Subsequently, at the age of 50–60, large areas of WMHs, lacunae and Cerebral microhemorrhage were often seen on imaging examination. Due to the high risk of complications in CADASIL patients, invasive procedures such as angiography should be avoided as much as possible to aggravate the disease progression (36). Since CADASIL is a non-atherosclerotic type of CSVD without amyloid deposition, we further summarized the characteristics of CADASIL, small atherosclerotic CSVD and MS (Table 1).

**Table 1:** Identification and comparison of CADASIL, small arteriolosclerosis CSVD and MS.

	**CADASIL**	**Small arteriolosclerosis CSVD**	**MS**
Clinical course	Progressive	Progressive	Multiphase course
Pathological manifestation	Deposition of osmiophilic particles in vascular smooth muscle	Small arteriosclerosis, lipohyalinosis, fibrinoid necrosis	Small perivenous inflammation
Paraventricular	Involved	The lesion is symmetrical and less likely to be adjacent to the ventricular canal	The lesion is asymmetric and often adjacent to the ventricular canal
Temporal pole	Early involvement with a characteristic lesion site	Rarely involved	Involved
Corpus callosum	Commonly involved	Rarely involved	Frequently involved
U-shaped fiber bundle	Involved	Not involved	Involved
Cortex	Not commonly involved	Not commonly involved	Involved
Optical nerve	Not involved	Not involved	Involved
Brainstem	Early stages are less frequently involved and late stages can involve	Central part of the pons	Peripheral part of the pons (dorsal and ventral), asymmetric
Spine	Not involved	Not involved	Focally located in the periphery of the spinal cord, short segments, posterior and lateral cords are common
Laboratory tests	Genetic testing	VRFs	OCB, IgG in the sheath

#### PACNS

PACNS is a kind of CSVD characterized by idiopathic vasculitis, which is more common in patients aged 50–60 years, and its lesions are limited to the white matter area of brain and spinal cord. There is no obvious specific clinical manifestation of this disease, and headache is the common first symptom in clinic (38). Laboratory examination can find that the levels of inflammatory markers and proteins in cerebrospinal fluid are increased. The pathological manifestations of PACNS include inflammation, intimal hyperplasia, small and medium artery occlusion and necrosis and others, which often involve pia mater and cortical vessels (39). Although there are no specific imaging features in PACNS, some imaging features including multiple cortical-subcortical infarction, hemorrhage, enhancement of brain parenchyma and pia mater also provide some evidence for clinical diagnosis of PACNS. Clinical routine angiography can sometimes find multiple stenosis of small and medium-sized vessels, but brain biopsy is the gold standard for the diagnosis of this disease (38, 40).

## MS

### Clinical Features of MS

MS disease pedigree includes radiologically isolated syndrome (RIS), clinically isolated syndrome (CIS), and clinically diagnosed MS. According to the different characteristics of the course of disease, the clinically diagnosed MS can be classified as follows: relapsing-remitting multiple sclerosis (RRMS); secondary progressive multiple sclerosis (SPMS); primary progressive multiple sclerosis (PPMS); progressive relapsing multiple sclerosis (PRMS). Among them, RRMS is the most common in clinic, while PRMS is the rarest (41).

### Pathological Features of MS

Generally, macular demyelination changes in white matter can be found in typical non-specific MS pathology. Classic MS lesions are considered to be slender or oblate lesions, usually distributed around venules (42). This demyelination lesion is thought to be associated with macrophage infiltration, perivascular cuff-like lymphocytes, accumulation of fibrin, and proliferation of reactive microglia. The white matter of MS patients is usually accompanied by abnormal deposition of hair iron, which is related to disease activity and usually deposited in the white matter where perivenous inflammation exists (43).

### MS Imaging Features

MRI is the most important tool to assist clinical diagnosis and disease monitoring of MS (44). McDonald criteria revised in 2010 have proposed the spatial and temporal multiplicity of MS lesions: spatial multiplicity requires white matter hyperintensity (WMH) in at least two of the four typical sites: paraventricular, juxtacortical, subtentorial and spinal cord; Multiple timing requires the presence of both enhanced and non-enhanced lesions in a single MRI scan or the discovery of new lesions in follow-up MRI (45, 46). During follow-up, the new white matter hyperintensity of T2 image and the white matter hyperintensity enhanced by contrast medium are related to the activity of inflammation; the average sustainable time of enhancement is about 3 weeks (47).

**Figure 2:**
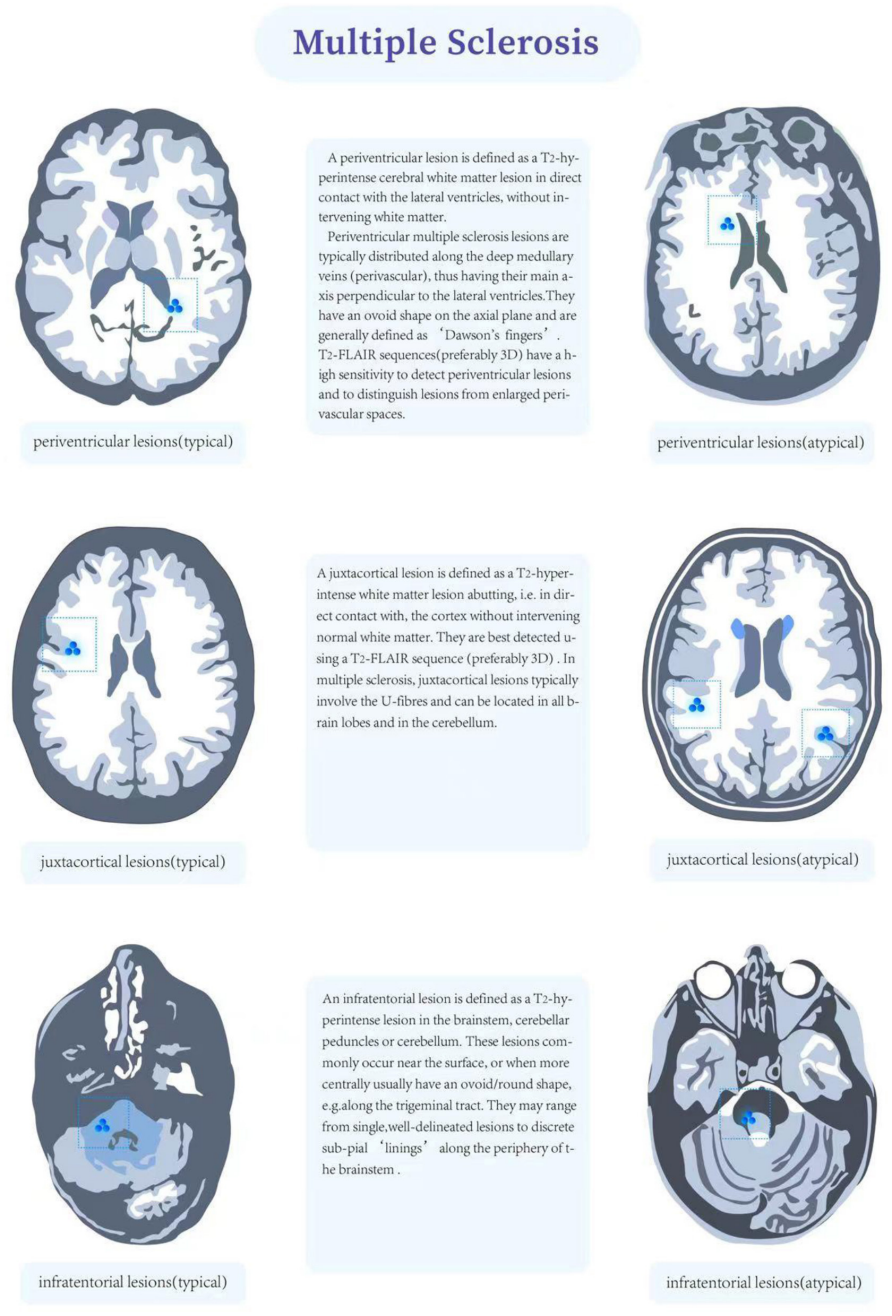
Charateristics of multiple sclerosis lesion that that are typical **(right)** and atypical **(left)**.

On MRI, Dawson fingers sign is a classic lesion shape of paraventricular WMH in MS patients, which is usually perpendicular to ventricle, oval and distributed around venules, adjacent to ependyma. This has certain significance for differential diagnosis with non-specific WMHs and CSVD. The lesions of CSVD often appear in the posterior horn of paracele, and similar lesions may appear in MS. In the early stage of MS, specific lesions can be found at the junction of corpus callosum and ependyma. These lesions have both local patchy changes and discrete distribution. We named this distribution feature as “dot-line sign,” which is usually easier to observe on sagittal T2-weighted imaging. This lesion is more likely to appear in the knee and body of corpus callosum (48). Of course, this kind of MS also needs to be differentiated from other types of diseases, including other primary or secondary demyelination diseases [neuromyelitis optica (NMO), acute disseminated encephalomyelitis (ADEM), CADASIL, Susac syndrome, progressive multifocal leukoencephalopathy (PML), etc.]; tumor diseases [lymphoma, glioma, gliomatosis cerbri (GC)-the latter is easily confused with leukoplakia due to its diffuse lesion]; traumatic injury (traumatic/diffuse axonal injury). The corpus callosum lesions of MS were enhanced in the acute phase while the corpus callosum lesions of CADASIL were not usually enhanced. Another typical lesion of MS is that WMHs near cortex involve U-shaped fibers under cortex. Generally, cortical lesions appear in the early stage of MS, which is related to cognitive dysfunction. Cortical lesions are usually smaller, and the contrast is more obvious under double inversion recovery sequence and high magnetic field intensity (49–51) (Figure 2).

### Summary of MS

The McDonald diagnostic criteria have suggested spatial and temporal multiplicity in the distribution of MS lesions: spatial multiplicity requires the presence of white matter hyperintensity in at least two of the four typical locations in the paraventricular, subcortical, subepicentral and spinal cord regions; temporal multiplicity requires the presence of both enhancing and non-enhancing lesions on a single MRI scan or new lesions on follow-up MRI (45, 46). New white matter hyperintensities on follow-up T2 image and contrast-enhanced white matter hyperintensities correlate with the activity of inflammation; enhancement can persist for an average of about 3 weeks (47). Dawson's finger sign is the classic lesion pattern of paraventricular white matter hyperintensity in MS patients; these lesions are usually perpendicular to the ventricles, ovoid in shape and distributed around small veins. They are contiguous with the ventricular canal, which is important in relation to the non-specific These lesions are usually perpendicular to the ventricles, ovoid and distributed around small veins (52). Early in MS, specific callosal lesions can be found at the junction of the corpus callosum and the ventricular canal, and these lesions These lesions are both localized and discrete, forming a subcallosal “dotted line sign” that is usually more easily observed on sagittal T2 weighted images in the sagittal plane are more easily observed. Such lesions are more likely to be found in the knee and body of the corpus callosum (48). In MS, the corpus callosum lesions are enhanced in the acute phase, whereas in CADASIL the corpus callosum lesions are usually not enhanced. Proximal cortical white matter hyperintensities in MS patients involve subcortical U-shaped fibers, which is also a classical lesion location. On the other hand, there is a growing awareness of cortical lesions in MS, which may have more prognostic and diagnostic value in the future. The prognostic and diagnostic value of these lesions may increase in the future. For MR spectroscopy, previous studies summarized that patients with MS have shown increased glutamate concentrations in acute MS lesions, indicating hypoxic condition (53–55).

McDonald criteria also included the subtentorial area. Among them, MS occurring in the brainstem has certain characteristics. The lesions are usually located in the periphery of the brainstem (CSVD brainstem lesions are usually located in the middle of the brainstem), and the lesions are asymmetric or located on one side of the brainstem with clear boundaries (56). Cerebellar lesions usually affect larger white matter structures, such as the pontine arm (middle-cerebellar peduncle), although middle-cerebellar peduncle and white matter in cerebellar hemisphere may also be affected. Spinal cord lesions are present in 30 to 40% of CIS cases and up to 90% of clinically confirmed MS patients (52).

MS also often involves cervical spinal cord, such patients are in dangerous condition, and even die in severe cases. Normally, the spinal cord lesions have a clear boundary, which is less than one or two spinal segments in longitudinal direction, <50% of the cross-sectional area of spinal cord in axial plane, and the spinal cord lesions are usually located in the white matter around the spinal cord. Extensive spinal cord swelling is rare. Active lesions may be enhanced, although less frequently than brain lesions. Spinal cord atrophy can be seen occasionally in long course and progressive MS, especially in the upper segment (57). The similar disease of MS in peripheral organs is chronic inflammatory demyelination polyradiculoneuropathy (CIDP), which is a chronic multiphase disease involving peripheral nervous system (PNS).

To sum up, the diagnosis of MS should be based on the comprehensive analysis of patients' clinical data, laboratory examinations and auxiliary examinations, including the analysis of patients' medical history, cerebrospinal fluid components, such as oligoclonal bands (OCB) and intrathecal immunoglobulin G (IgG) synthesis, abnormal visual evoked potential (VEP) and imaging analysis. However, MS is often misdiagnosed due to atypical imaging features. About 25% of patients received MS treatment without suffering from MS. The imaging features of CSVD, NMO and ADEM are often confused with MS (58), but there is still no effective means to distinguish them, which poses great challenges for clinical diagnosis and treatment.

## Relationship Between CSVD and MS

Currently, it is thought that there may be interaction between CSVD and MS, because the progression of CSVD and MS is positively correlated with age. Advanced age means that glial cells may have hypoxia (59), mitochondrial dysfunction (60), iron deposition (61) and other dysfunctions. These changes are obviously related to neurodegenerative changes. Recently, Geraldes et al. summarized that CSVD shares some features with MS and has been shown to contribute to the neuronal damage seen in vascular cognitive impairment (62). In addition, CSVD (35) has been found to be associated with neurodegeneration in young people with VRFs (63), which may also promote age-related neurodegeneration in MS. Therefore, we summarized the main possible reasons for the interaction value between MS and age-related CSVD: (i) the life expectancy of MS patients is longer (64), over 60 years old (65), and the risk of vascular complications is higher; (ii) vascular complications can promote the disease progression of MS (66), shorten the expected survival time (67–69), aggravate WML load and brain atrophy (70); (iii) the focal demyelination lesions in the watershed area of MS (71) are characterized by hypoperfusion and hypoxia (72–74), suggesting that there is an important relationship between the pathology of MS and cerebral artery perfusion. In addition, Lucchinetti et al. previously reported MS lesions divided into four patterns, in which two patterns (I and II) showed close similarities to T-cell-mediated or T-cell plus antibody-mediated autoimmune encephalomyelitis, and the other patterns (III and IV) were highly suggestive of a primary oligodendrocyte dystrophy, reminiscent of virus- or toxin-induced demyelination rather than autoimmunity. The pattern III is distal oligodendrogliopathy which means oligodendrocytic apoptosis due to hypoxia or viral infection (75, 76). Similar observations are reported independent researchers (77, 78). Santiago Martinez Sosa et al. also reported that The deep and periventricular white matter is preferentially affected in several neurological disorders, including CSVD and MS, suggesting that common pathogenic mechanisms may be involved in this injury and considered the potential pathogenic role of tissue hypoxia in lesion development, arising partly from the vascular anatomy of the affected white matter (1); (iv) chronic inflammatory microenvironment of MS can promote CSVD, furthermore, Robin et al. found that shared mechanism of white matter damage in ischemia and MS and reported that inflammation acts in distinct pathways because of the differing nature of the primary insult (79); (v) some MS patients benefit from using drugs targeting microvascular system (80–82). Furthermore, Chitnis et al. reported a case of Balo's concentric sclerosis, a variant of MS, with CADASIL mutation and suggested systematic testing for the CADASIL mutation in patients with a demyelinating presentation consistent with Balo concentric sclerosis or significant restricted diffusion on MRI (83) (Table 1).

The influencing mechanism of CSVD on the imaging features of MS remains to be unclear. It is possible that the increased load of T2 phase lesions in MS patients with VRFs is due to the superposition effect of paraventricular vascular WML, lacuna and microhemorrhage. Similarly, the more severe brain atrophy associated with VRFs in MS patients may also be due to the superposition of VRFs on MS basis (84). Although the previous understanding of MS is that multiple demyelination areas are found in pathological sections, and axons in these areas are relatively preserved, imaging and pathological studies have found that neuron/axon damage can occur at an early stage and is related to active inflammatory activity and demyelination (85, 86). However, age and duration of disease can affect the inflammatory response of WM and gray matter (GM) in lesion and non-lesion areas of MS, and the inflammatory response seems to decrease in elderly patients with longer course of disease (87, 88). In these patients, neurodegeneration may be related not only to persistent low-intensity inflammatory activity, but also to various mechanisms such as energy deficiency, oxidative damage, hypoxia, and energy reserve failure (72). Energy failure is an important concept for MS pathogenesis. Glucose and lactate transporters and connexin gap junctions have a crucial roles for energy transport among glial cells in MS (89–93). Recently, Philips et al. summarized oligodendrocytes transfer energy metabolites to neurons through cytoplasmic “myelinic” channels and monocarboxylate transporters, which allow for the fast delivery of short-carbon-chain energy metabolites like pyruvate and lactate to neurons. These substrates are metabolized and contribute to ATP synthesis in neurons (93). But this deficient process may be a cause of pathogenesis for MS. Age-related pathological processes, such as AD or vascular disease, may amplify the effects of these mechanisms and lead to increased neuronal damage (87).

The presence of CSVD in MS patients may mean an additional blow to the compensatory mechanism. In this case, chronic hypoxia caused by vascular dysfunction and hypoperfusion can cause neuron death and aggravate neurological dysfunction. In neurodegenerative diseases such as AD (94, 95) and vascular cognitive impairment (96), cerebral vascular lesions promote neuronal damage, aggravate pericyte and astrocyte dysfunction caused by chronic hypoperfusion and BBB permeability changes, oxidative stress, inflammation and mitochondrial damage (97). The theory of “blood vessel-neuron-inflammation” centered on NVU dysfunction in neurodegenerative diseases is also applicable to MS (95). Energy deficiency and tissue hypoxia caused by mitochondrial dysfunction in MS will lead to ion disorder and axonal degeneration, and the accompanying CSVD may aggravate this process, especially in watershed area (72). Not only the lesions related to MS and arteries and veins need further study (72), but also the characterization and quantification of CSVD, including the score of arterial wall changes, microbleeding and microinfarction, which is conducive to further exploring the relationship between CSVD and MS. Moreover, Geraldes et al. based on post-mortem study reported that an excess burden of cerebral small vessel disease in multiple sclerosis may explain the link between vascular comorbidity and accelerated irreversibility disability (98).

## Summary

In conclusion, this review compares and summarizes the clinical, pathological and imaging features of MS and CSVD and the effect of vascular diseases on MS, which is conducive to deepening our understanding of these two diseases, making correct differential diagnosis and giving patients correct treatment strategies in time.

## Author Contributions

BW, HaoL, and XL contributed to the conceptualization of the manuscript. BW, HaoL, XL, XH, LG, RF, KangC, and WW contributed to the writing of the manuscript. XH, HaiL, KangnC, ZZ, and LX contributed to the editing and revising of the manuscript. All authors contributed to the article and approved the submitted version.

## Conflict of Interest

The authors declare that the research was conducted in the absence of any commercial or financial relationships that could be construed as a potential conflict of interest.

## Publisher's Note

All claims expressed in this article are solely those of the authors and do not necessarily represent those of their affiliated organizations, or those of the publisher, the editors and the reviewers. Any product that may be evaluated in this article, or claim that may be made by its manufacturer, is not guaranteed or endorsed by the publisher.
